# Universal Count Correction for High-Throughput Sequencing

**DOI:** 10.1371/journal.pcbi.1003494

**Published:** 2014-03-06

**Authors:** Tatsunori B. Hashimoto, Matthew D. Edwards, David K. Gifford

**Affiliations:** 1 Computer Science and Artificial Intelligence Laboratory, Massachusetts Institute of Technology, Cambridge, Massachusetts, United States of America; Heinrich Heine University, Germany

## Abstract

We show that existing RNA-seq, DNase-seq, and ChIP-seq data exhibit overdispersed per-base read count distributions that are not matched to existing computational method assumptions. To compensate for this overdispersion we introduce a nonparametric and universal method for processing per-base sequencing read count data called Fixseq. We demonstrate that Fixseq substantially improves the performance of existing RNA-seq, DNase-seq, and ChIP-seq analysis tools when compared with existing alternatives.

## Author Summary

High-throughput DNA sequencing has been adapted to measure diverse biological state information including RNA expression, chromatin accessibility, and transcription factor binding to the genome. The accurate inference of biological mechanism from sequence counts requires a model of how sequence counts are distributed. We show that presently used sequence count distribution models are typically inaccurate and present a new method called Fixseq to process counts to more closely follow existing count models. On typical datasets Fixseq improves the performance of existing tools for RNA-seq, DNase-seq, and ChIP-seq, while yielding complementary additional gains in cases where domain-specific tools are available.

This is a *PLOS Computational Biology* Methods article.

## Introduction

High-throughput sequencing is used in a variety of molecular counting assays [1] to study protein-DNA binding, transcription, and the dynamics of chromatin occupancy. ChIP-seq [2], used to study protein binding to the genome, captures short DNA fragments that are attached to a protein of interest after a chemical treatment that affixes proteins to nearby DNA molecules. High-throughput sequencing of these DNA fragments, followed by identifying their originating location in the genome, allows for the identification of read-enriched areas. These enriched regions correspond to locations where the protein of interest was bound, perhaps indirectly, to the DNA. RNA-seq [3], [4], used to study gene expression, requires isolating the RNA content of a sample, converting it to DNA, and sequencing the resulting DNA library. Mapping or assembling the DNA reads and assigning them to exons or transcripts enables the genome-wide quantification of gene expression. DNase-seq [5], [6] identifies regions of open chromatin and patterns of transcription factor binding by employing an enzyme that preferentially cuts DNA in accessible positions. Retrieving and sequencing the resulting library of DNA fragments, followed by identification of the originating locations, allows for a genome-wide characterization of chromatin occupancy. A unifying task in these analyses is comparing read count profiles, obtained from read mapping results, across varying biological samples or experimental conditions.

Although a myriad of specialized methods exist for analyzing read count data, it is frequently assumed (implicitly or explicitly) that read counts are generated according to a Poisson distribution with a local mean. The assumption is explicitly introduced by using the Poisson density directly as well as implicitly by relying on binned per-base counts in ranking and statistical testing (see Text S1). When read count data exhibit overdispersed per-base read distributions, a Poisson model may produce erroneous or noisy results. This occurs because the data are not matched to the modeling assumption, resulting in incorrect assessments of statistical significance. While it is well known that the distribution of per-base counts within a single experiment is in fact typically overdispersed, there has not been a precise characterization of the degree of overdispersion and its effects on downstream analysis for general sequencing data.

We introduce a general and asymptotically correct preprocessing technique called Fixseq for correcting per-base and per-experiment read counts. Fixseq reduces noise and increases stability in subsequent inference procedures and complements existing literature on applications of heavy-tailed distributions [7]–[9]. Existing literature for preprocessing focuses on either de-duplication, which removes all but one read per base, or normalization techniques for RNA-seq data, which generally operate over exon-level counts. We have previously dealt with these problems when developing a ChIP-seq caller with adaptive count truncation [10] and found that this was effective in practice (see Text S1 and Table S1), but this work aims to construct a more general preprocessing scheme that works for any method and sequencing assay.

The normalization strategy of de-duplication is prevalent in multiple ChIP-seq peak callers [11], but less common in RNA-seq data analysis where highly-expressed transcripts may be expected to have many duplicate reads. However, a handful of RNA-seq processing algorithms remove duplicates as a conservative choice to avoid nonlinear PCR amplification errors [12], [13].

Existing RNA-seq normalization techniques work at a higher conceptual level than Fixseq, using information from a local sequence context to correct exon- or transcript-level sums and reduce the impact of confounding noise covariates [7], [14]–[18]. In our RNA-seq results we show that Fixseq can enhance the results of methods such as DEseq that already account for exon-level overdispersion as well as provide complementary information to methods that correct for mappability and GC content (see Figure S2). While these methods are valuable for RNA-seq and binned count statistics, they are less applicable to other sequencing data types and often have specific modeling assumptions that rely on the mechanisms of transcription, cDNA library preparation, and DNA sequencing. Covariate-based normalization techniques designed for other assays, like ChIP-seq, require identified binding sites as a prerequisite and are designed to correct only windowed read counts [19], rendering them unusable as preprocessing tools for algorithms which require per-base count data. Other assay-specific normalization tools (e.g. [20]–[23]) require extensive domain knowledge and application-specific modeling strategies and, while valuable, must be developed independently for each new assay.

In contrast to most of these existing strategies, Fixseq works at a lower and more general level, the per-base count, and attempts to decrease the false positive rate rather than recover lost signal caused by sequencing artifacts. This approach is applicable to many types of sequencing assays and downstream processing algorithms, without additional assumptions. This universal nature allows for Fixseq to be applied to any type of sequencing count data, without training phases or specialized model-building. However, in cases where applicable covariate-based or assay-specific normalization tools exist, they may be used in addition to Fixseq in order to leverage complementary gains (as in Figure S2). One additional consequence of our work is a generalization of the de-duplication heuristic into a broader and asymptotically correct preprocessing technique.

## Results

### Read counts in sequencing data are highly overdispersed

**Figure 1:**
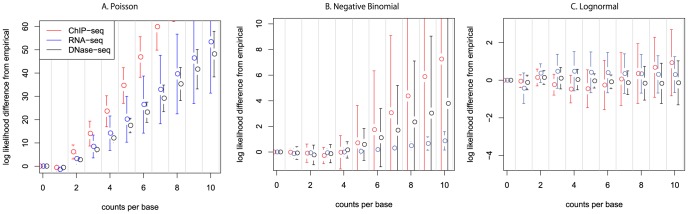
Log-likelihood difference between maximum likelihood and observed distributions. Differences in log-likelihood per base between the fitted model and the empirical distribution, also interpreted as the log-difference between observed and fitted counts. This error metric represents the error when calculating p-values or significance tests using a Poisson assumption. Three assay types are shown in each panel, analyzed by three models: (a) Poisson. (b) Negative binomial. (c) Log-normal Poisson. A model that fits the data would have points along the 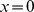 line. Consistent deviation from zero by all distributions show that none of the distributions fit all assays well.

**Figure 2:**
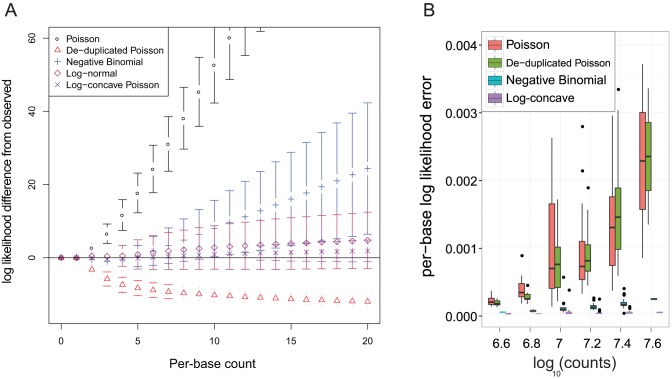
Log-likelihood errors for ENCODE ChIP-seq data. The distribution described as log-concave is the statistical model used in Fixseq. Subfigure (a) shows that de-duplication Poisson can control high per-base errors much like overdispersed models, but Subfigure (b) shows that de-duplication error rises rapidly as sequencing depth increases.

**Figure 3:**
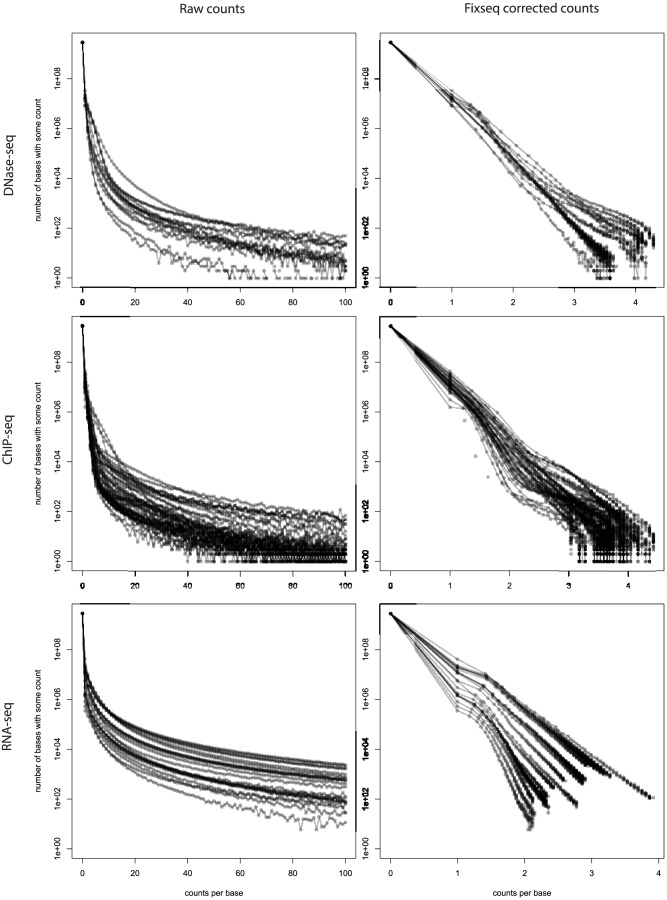
Distribution of counts for 14 DNase-seq experiments, 23 RNA-seq experiments and 87 ChIP-seq experiments. The left panel shows uncorrected counts, and the right shows counts after correction. Poisson distributed counts would follow a straight line; all experiments show significant deviation from linearity that is corrected by Fixseq.

The distributions of per-base mapped read counts in all ChIP-seq and RNA-seq runs for the human embryonic stem cell type (H1-hESC or ES cells) in the ENCODE project and a set of K562 cell line DNase-seq experiments (see Text S1) show evidence of consistent and significant overdispersion (Figures 1, 2, and 3). This extra variation arises from a myriad of biological and technical sources, including true variation in factor binding signal or expression levels genome-wide, variation in molecular sequencing affinity, and variation in read mapping accuracy. The overdispersion we find is complex and cannot be directly categorized as the result of a well-known parametric distribution such as gamma-Poisson [24], [25] or log-normal-Poisson [26] (Figures 1 and 2).

We quantified the degree of overdispersion with respect to a distribution by comparing per-base empirical log-likelihoods against the per-base maximum log-likelihood distributions for the Poisson, negative binomial, and log-normal Poisson, where the per-base rates of the Poisson are assumed to be drawn from a log-normal distribution. For the negative binomial and log-normal Poisson, maximum likelihood distributions were found via numerical optimization with randomized restarts.

The deviation from Poisson is consistent across experiment and assay type, as shown in the left column of Figure 3. We would expect a completely linear histogram of log-counts but actually observe significant overdispersion, shown by large number of high-count bases.

Figure 1 shows that none of the parametric distributions we tested fit the observed counts well. The Poisson significantly underestimates the number of bases with more than one read, with the probability of having ten counts land on the same base estimated to be 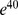 times less than the observed number of counts. The negative binomial has previously been shown to be effective for modeling exon-level RNA-seq data, and we confirm that negative binomial fits per-base RNA-seq data well. However, we find that it fails to capture the wide variation in overdispersion for ChIP-seq, underestimating the high count bases by at most a factor of 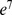. The log-normal Poisson fit shows that real-world sequencing data is not simply heavy-tailed; it has heavy tails whose shapes are dependent on the assay type. The log-normal Poisson is traditionally considered an extremely heavy-tailed distribution, and while it is relatively correct for ChIP-seq, it significantly overestimates the tail mass for DNase-seq experiments.

The wide variation in overdispersion level and type suggests that any single parametric approach is unlikely to be effective for all assay types. Instead of attempting to model each assay type with a separate parametric family, we will use nonparametric distributions that are flexible enough to fit all observed assay types well.

### Count correction via data transformation

While we have already seen that the Poisson assumption fails for most of the assay types we consider, it is not feasible or necessary to modify every analysis algorithm to use overdispersed distributions. Instead, for the class of inference algorithms which implicitly or explicitly assume Poisson counts and independent bases, such as most ChIP-seq callers, DNase-binding identifiers, and RNA-seq exon read counting methods, we can construct improved datasets with transformed counts that correct for overdispersion.

The two major nonparametric approaches to data transformation are quantile normalization, which matches input samples to a reference sample via their quantiles, and distribution matching, which fits a distribution to both the input and reference and constructs a mapping function between them.

Quantile normalization, which is a popular approach in the microarray literature, cannot easily be adapted to sequencing data, due to the large number of bases with equal counts. In order to rank normalize our observed counts to a Poisson, we would have to arbitrarily break ties between bases with equal reads, which could lead to spurious inference as well as force bases with non-zero counts to be discarded.

Instead of breaking ties, we employ a different approach to distribution mapping: given a distribution 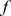 over counts, we find the mapping which makes the density of the Poisson equal to that of the given distribution 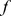. By fitting a distribution 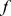 to the observed counts, we avoid the problem of tied counts and allow for a continuous mapping. One advantage of viewing this approach under a distribution mapping framework is that it allows us to understand the theoretical basis of the de-duplication heuristic that is a popular preprocessing method for ChIP-seq data.

Our approach transforms the non-Poisson, curved count histogram on the left column of Figure 3 into the Poisson-like linear count histogram on the right column of Figure 3.

### De-duplication acts as a degenerate data transform

De-duplication, or removal of all but one read at each base position, has gained adoption in the ChIP-seq analysis literature as an effective way of reducing noise and improving replicate consistency [27], [28]. ChIP-seq event callers such as MACS [29] and SPP [30] either de-duplicate by default or strongly suggest enabling de-duplication.

The heuristic of de-duplication can be derived as a distribution mapping data transformation by assuming that the read counts arise from a degenerate count distribution, where the number of bases with non-zero reads is drawn from a binomial, and the number of reads at non-zero bases is drawn from a uniform noise component over 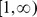. In this case, the probability of all non-zero counts are equal, and they should be mapped to the same value after the transform. Conversely, any data transform preserving rank order will not fully de-duplicate but will instead monotonically re-weight counts.

De-duplication works well in practice by drastically reducing the error and additional variance from overdispersion, despite assuming that the data follow a degenerate distribution. Figure 2a shows the performance of various overdispersion correction methods using the per-base log-likelihood error for the Poisson, de-duplicated Poisson, negative binomial, and the log-concave Poisson distribution, which we use in Fixseq. These per-base errors reflect the expected error in statistical significance testing under window-based DNase-seq and ChIP-seq callers, as errors in the log-likelihood propagate directly into error in the Poisson test statistic.

Preprocessing by de-duplication does not continue to reduce per-base errors as sequencing depth increases. Figure 2b shows that the log-likelihood error per-base increases rapidly as a function of the total sequencing depth. The Poisson and de-duplicated Poisson both have average per-base error which increases similarly as a function of sequencing depth. This confirms the observation that as sequencing depth increases, more of the mappable genome will have at least one mapped read, leading to a loss of predictive power.

Therefore while de-duplication may be effective at lower sequencing depths, it relies upon a limited heuristic justification and will not remain effective as sequencing depths increase. On the other hand, Fixseq significantly outperforms de-duplication at modeling the observed data distribution as sequencing depth grows (Figure 2b) and is asymptotically consistent under relatively weak assumptions.

We compared three methods of count preprocessing: original (raw) counts, removal of all duplicates (de-duplication), and our novel preprocessing technique (Fixseq). These preprocessing schemes are compared across three assay types and in multiple experiments and in multiple contexts. We show that Fixseq consistently improves performance, with substantial improvements obtained in certain cases.

### DNase-seq

We evaluate our model on the ability to identify transcription factor binding sites based upon DNase-seq counts on the ENCODE human K562 DNase-seq data using two different methods: an unsupervised task using the CENTIPEDE binding site caller [31] and a supervised task using a linear classifier. The binding site predictions are compared against all matching ChIP-seq calls for the same factor on a matched cell type, and we evaluate the algorithm on the fraction of ChIP-seq calls we are able to recover. The details of the comparison, such as the PWM matching and cutoffs, follow the techniques used by CENTIPEDE.

**Figure 4:**
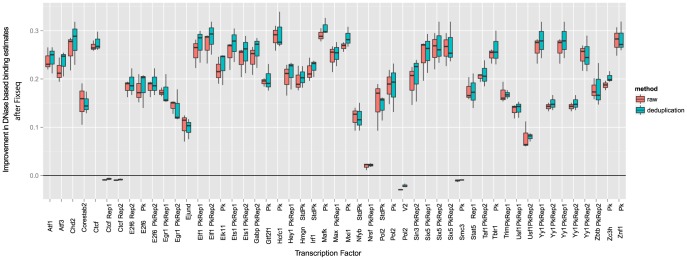
AUC comparisons for baseline methods compared to Fixseq. Boxplots depicting AUC improvement across multiple factors (boxes above zero represent improvement due to Fixseq). Variance was estimated using one thousand bootstrap replicates each. Transcription factors with no significant difference across methods (p = 0.05) are not shown. Of the 301 factors tested, 90 could be predicted nontrivially (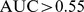) via CENTIPEDE. Of these, 51 show significant differences.

In the unsupervised task shown in Figure 4, Fixseq shows small but consistent improvements on nearly all runs and all methods, and on many factors we show improvements up to a 0.3 increase in area under the curve (AUC), a metric of accuracy. These large performance increases indicate that Fixseq rescued an otherwise failed run.

### ChIP-seq

We tested Fixseq on 87 ES cell ChIP-seq experiments from the ENCODE project [32], using the ChIP-seq callers MACS [29] and PeakSeq [33] on original counts, de-duplicated counts, and Fixseq processed counts with rounding (see Text S1). Following prior work in evaluating ChIP-seq caller accuracy [28], [34], we selected two evaluation criteria: replicate consistency of q-values and the number of overlapping ChIP-seq events across replicates.

**Figure 5:**
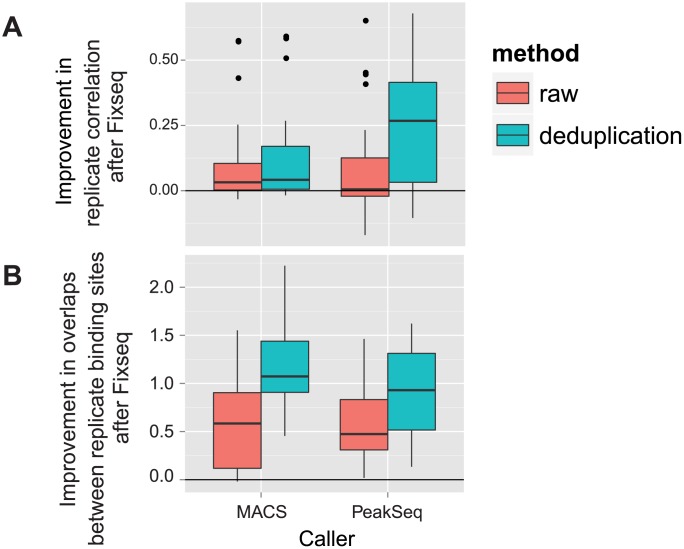
Event reproducibility for ChIP replicates. Subfigure (a) shows that Fixseq increases the q-value correlation between replicates. Subfigure (b) shows the increase in the log number of bases overlapping between calls on replicates due to Fixseq. Fixseq calls have consistently higher peak overlaps between replicates for both MACS and PeakSeq.

We evaluate quantile-quantile correlation for replicate consistency, as this allows us to evaluate the distribution of q-values generated by each method without pairing binding sites explicitly. The quantile-quantile (QQ) correlations are an effective means of detecting not only whether we call similar numbers of binding sites across replicates, but also whether our ChIP-seq call confidence is consistent across replicates. The quantile-quantile correlations across all analyzed ENCODE ChIP-seq experiments shown in Figure 5a strongly suggest that Fixseq stabilizes the distribution of q-values from PeakSeq and MACS. Fixseq outperforms both raw counts and de-duplication for PeakSeq and improves on de-duplication significantly for MACS (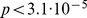).

An alternative measure of ChIP-seq experiment quality is the number and size of overlapping sites across replicates. Fixseq increases the number of overlapping sites in both methods, showing that Fixseq improves consistency of localization of sites as well as the ranking of ChIP-sites (Figure 5b).

### RNA-seq

We ran Fixseq on all 23 ES cell RNA-seq datasets from ENCODE and evaluated the replicate consistency of the original read counts, de-duplication, and Fixseq. Using the ENCODE alignments, we followed the analysis technique suggested by DEseq [7] and mapped reads and adjusted counts to exons and generated exon-level counts.

Replicate consistency was measured in two ways: Spearman's rank correlation and the number of false positive differential expression events called by DEseq [7] across replicates. Spearman's rank correlation on exon counts was chosen to characterize the run-to-run variability between replicates, while DEseq was chosen to represent Fixseq's ability to enhance existing techniques that attempt to handle exon-level overdispersion.

**Figure 6:**
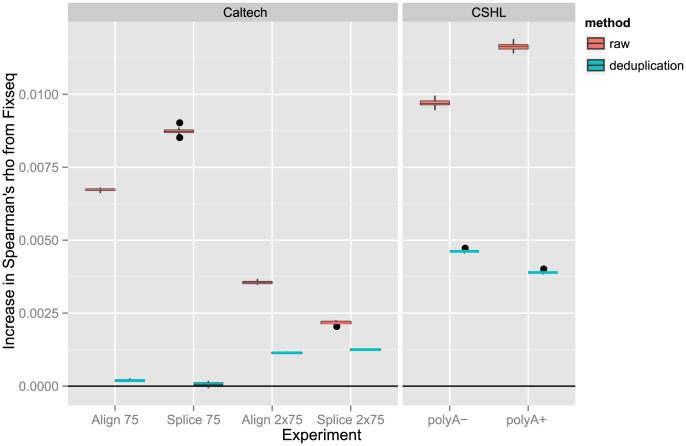
Bootstrapped differences in Spearman's rank correlation coefficient for ENCODE RNA-seq experiments. Higher rank correlations across exon expression measurements between replicates indicate greater data quality and reproducibility. Fixseq increases the rank correlation coefficient for almost all experiments, though the improvements compared to de-duplication for the CSHL 75bp align and splice experiments are minimal.

The rank correlation between replicates shown in Figure 6 is significantly higher for Fixseq processed counts than for both raw counts and de-duplication (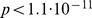). We make even greater improvements in the 75-bp single-end RNA-seq datasets, where it is possible that difficulty or ambiguity in read mapping causes single-base spikes that adversely affect replicate consistency. The RNA-seq correlations also support our earlier claims that de-duplication performance will begin to degrade as sequencing depth increases. In both the paired-end Caltech and Cold Spring Harbor Laboratory (CSHL) experiments we find that the original counts are on average better than the de-duplicated counts due to the higher coverage per base.

Our DEseq results in Table 1 are consistent with the correlation results, with Fixseq calling fewer differential exons across replicates despite being a less aggressive truncation scheme than de-duplication. Following the DEseq analysis pipeline, we used replicates to estimate exon-level overdispersion and identified exons differential across replicates at 0.05% FDR. The number of exons called differential across replicates in Table 1 are consistently the lowest for Fixseq out of all methods tested. Since Fixseq is a shrinkage method, we would expect there to be fewer false positives between replicates under Fixseq than the original counts. For example, if we preprocess by deleting all counts, we would have a trivial zero false positive rate. However, this is likely not the method by which Fixseq decreases false positive rate since we outperform de-duplication, which is an even more aggressive shrinkage method. This suggests that the counts we retain are consistent between replicates.

**Table 1:** Differential expression results across replicate experiments.

	Caltech Align 75	Caltech Splice 75	Caltech Align 75x2	Caltech Splice 75x2	CSHL polyA-	CSHL polyA+
Original	2903	1454	5719	2748	8403	6955
De-duplication	2559	1033	4951	2027	6640	5253
Fixseq	**2213**	**944**	**4230**	**1989**	**5319**	**4374**

Number of exons falsely called differentially expressed between biological replicates by DEseq at 5% FDR level; entries with the fewest false calls are bolded. There are 256324 total exons in the annotation set.

## Discussion

We have shown that per-base count overdispersion is a widespread and consistent phenomenon in high-throughput sequencing experiments. While correcting for exon-level overdispersion has been studied in RNA-seq, per-base methods and corresponding tools for ChIP-seq and DNase-seq have largely been unexplored outside of aggressive count truncation methods particular to individual algorithms. One reason for the slow adoption of overdispersed models has been the empirical success of the de-duplication heuristic as a preprocessing scheme. However, we show that de-duplication assumes the data arise from a degenerate distribution, and that the performance of de-duplication will degrade as sequencing depth increases.

Fixseq corrects overdispersed sequence count data by assuming that the data arise from a flexible class of log-concave distributions. We designed a novel and fast inference algorithm for the class of Poisson log-concave distributions as well as effective rounding schemes. In a diverse array of validation tasks, including DNase-seq binding site identification, ChIP-seq peak calling, and RNA-seq self-consistency, Fixseq consistently increased performance compared to both original counts and de-duplication. In cases where domain-specific correction schemes exist, Fixseq can operate in conjunction with them to yield complementary gains. While not replacing other sophisticated methods that can model the intricate biological realities of a new sequencing assay, Fixseq aims to provide a useful solution for all count-based sequencing assays without modification for new protocols.

The Fixseq method has the potential of broadly improving inference for high-throughput sequencing by bringing sophisticated overdispersion correction to a large number of existing analysis pipelines, while being applicable to future assays without lengthy development and modeling cycles. Additionally, the modeling and inference results we presented can be used in new flexible analysis procedures for count data.

## Methods

Our count preprocessing method, Fixseq, consists of three components:

1. Parameter inference for a novel class of distributions called log-concave Poisson distributions.
2. A probability integral transform method to map counts generated under log-concave Poisson to a Poisson distribution.
3. Rounding techniques to adapt datasets to methods that utilize only integral counts.

In the case that the algorithm downstream of our method is able to take weighted counts, Fixseq inherits all the favorable properties of the maximum likelihood estimator (MLE) and can guarantee unbiased and asymptotically consistent inference under the assumptions of per-base independence and log-concave count distributions.

### Poisson log-concave distributions

The challenge of constructing a universal preprocessor is finding a class of count distributions that is flexible enough to model a variety of assay types while remaining non-degenerate. We achieve this goal by letting the per-base rates of a Poisson distribution be drawn from a nonparametric class of distributions called log-concave. Log-concave distributions are a family of distributions 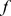 for which the log-density is a concave function. This allows us to write any log-concave function in terms of 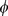, a concave function:
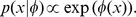



The log-concave family includes a large family of unimodal distributions, such as most of the exponential family (including common cases such as the normal, gamma with shape parameter greater than one, Dirichlet) [35]. Important exceptions include all multi-modal distributions and distributions with super-exponential tails such as the 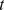-distribution or the Cauchy.

In sequencing experiments log-concave Poisson families are capable of modeling zero-inflation as well as mixtures induced by copy number variation for low Poisson rates with 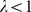, where the overall distribution remains unimodal. If such distributions are needed, straightforward extensions for mixtures of log-concave distribution are well known [36].

Our algorithmic contribution is the use of compound log-concave distributions, where we use latent log-concave distributions which generate Poisson counts along the genome. Inference for latent log-concave distributions does not follow directly from recent results in log-concave density estimation because of the ambiguity of parameters in the latent space.

The full model is as follows: per-base counts 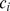 are generated by per-base log-rates 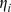, which are drawn from a log-concave distribution with density 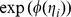:
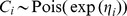


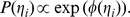



Note that the two exponential operators above are intentional: 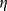 is a log-rate and therefore is exponentiated to become the Poisson rate, while 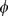 is a log-density and therefore is exponentiated to create an unnormalized density 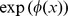.

The form of this model naturally suggests an expectation-maximization strategy, which has been shown to be effective for clustering tasks [37]. However, while we can perform expectation maximization using numerical quadrature, we find in practice that the algorithm is unstable and converges extremely slowly.

Instead we propose an inference technique based upon accelerated proximal gradient descent. The marginal likelihood for counts can be written as:
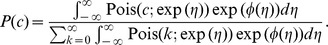



The bottom term normalizes the log-concave distribution. Approximating the integral with a sum over 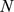 quadrature points 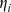 we obtain:
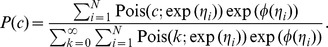



Since 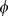 is always evaluated at the fixed points 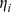, we can use the shorthand 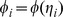 and let 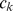 be the number of bases with 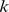 counts. Then the maximum likelihood estimator for the observed data is given by:
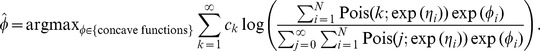



Both the objective function and constraints are concave, and therefore we can use accelerated gradient descent to quickly find the global optimum [38]. In particular, we use a method called proximal gradient descent, which optimizes a objective function of the form 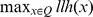 by repeatedly applying:
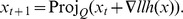




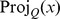
 is defined as the projection of 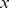 onto 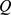.

Our gradient, 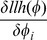, is easily written in terms of the shorthand, 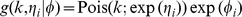, as:
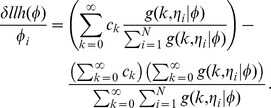



This gradient has a straightforward interpretation: the first term is the distribution of 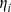 when observing the counts 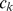 and the second term is the distribution of 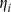 predicted from the prior 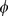 alone. The gradient works to minimize the difference between these observed and prior terms.

The projection operator 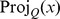 taking 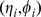 and producing the closest concave 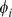 is the well-known concave regression algorithm [39].

The inference algorithm is guaranteed to converge to a global optima of the quadrature approximation, which as the number of quadrature points increase will converge to the global optima. If there are sufficiently many quadrature points, Fixseq will converge to the log-concave distribution closest to the data-generating distribution in the KL-divergence sense [37]. For the results, we use one million quadrature points throughout.

When compared to the naive expectation maximization based method, our algorithm converges more quickly, with average runtime on our DNase datasets reducing from 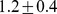 hours per dataset for EM down to 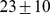 minutes for the gradient based method on a standard laptop with Intel i7 2.5ghz, with a slight increase in goodness of fit for the gradient approach.

### Count adjustment via probability integral transform

Once we fit a log-concave distribution 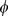, we need to be able to convert counts generated under the log-concave Poisson into those generated by the continuous extension of the Poisson. We will define the transformation from raw counts to processed counts via the probability integral transform.

Throughout this section, we will use the continuous extension of the Poisson PDF, CDF and the analogous densities for the log-concave compound distributions, defined below as:
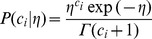


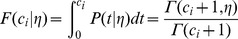


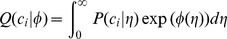


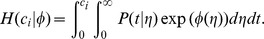



Given the continuous extensions, we can apply the probability integral transform directly. Given 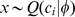, we can generate a uniform random variable 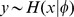, from which we define the Poissonization transform, 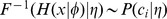. Since we are applying the probability integral transform to the continuous extensions of the Poisson, we are not guaranteed integral counts or consistency properties generally implied by the probability integral transform. However, for our purposes it is sufficient that the quantiles and densities are matched.

Our preprocessing function 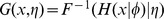 takes any 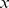 distributed as Poisson-log concave with latent distribution 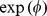 and returns adjusted counts distributed as Poisson. This operation preserves all of the joint structure of 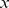 and acts as a black box which exchanges the Poisson assumption used in a method for a compound Poisson log-concave distribution assumption. Alternatively, one can consider using 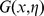 to be a re-weighting operation, which ‘fixes’ the underestimated tail density of the Poisson.

Examples of the 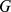 function for various ENCODE assays are shown as Figure S1.

Finally, 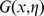 contains a free parameter 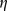 which we can choose freely. While any 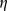 would be essentially equivalent, we choose to set 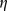 to be the median of the latent density throughout our results.

### Rounding schemes

While some algorithms, such as CENTIPEDE [31] for DNase-seq binding, can take weighted (fractional) read counts, many existing algorithms will only accept integral counts. We therefore develop two rounding schemes that can improve performance while providing integral counts.

The straightforward count flooring schemes, where 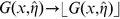 can be thought of as generalizations of de-duplication. In a typical DNase-seq experiment with 100 million reads, we find that flooring results in bases with 5 counts or less being de-duplicated, and those with 6 or more being reduced to two reads per base. While in the low-count cases, flooring is nearly identical to de-duplication, as sequencing depth increases, we expect our floored preprocessor to begin strongly outperforming de-duplication.

We also propose a more sophisticated randomized rounding scheme, where we take 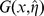 and let 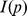 be a Bernoulli random variable with probability 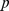, then the randomized round scheme generates simulated datasets whose counts round either up or down by the proximity of the adjusted count to its neighboring integers:
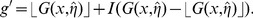
(%)


**Figure 7:**
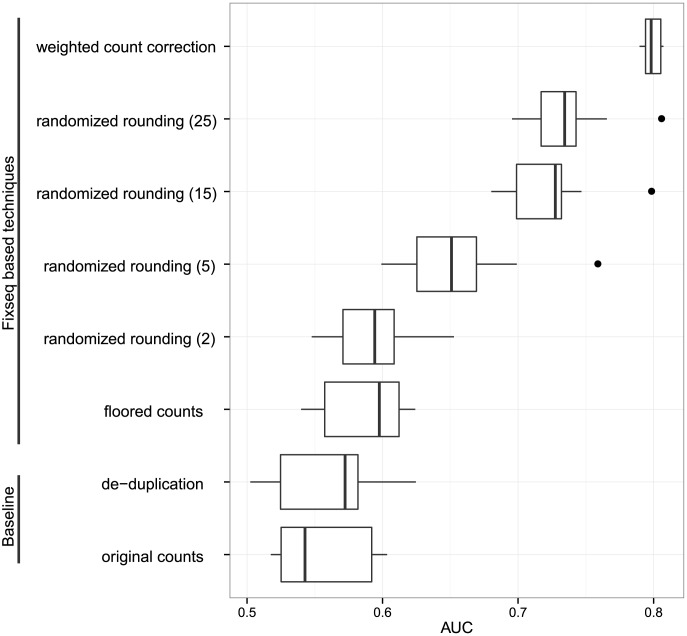
Performance comparison of rounding schemes on unsupervised DNase binding prediction. All rounding schemes outperform baseline methods (bottom left) but only randomized rounding approaches performance of the weighted counts (top right).

We compared these schemes on DNase data, where the unsupervised classifier, CENTIPEDE, was capable of accepting weighted counts, allowing us to compare various rounding schemes to the direct weighting scheme using the same comparison method as our DNase-seq results. The results in Figure 7 show that floored counts provides a statistically significant, but similar, performance to de-duplication and randomized rounding strictly improves upon both schemes. Rounding is relatively dependent on the number of randomly-sampled replicates, with around thirty samples needed to achieve its peak performance. The peak performance achieved by weighted counts is not achievable by any rounding scheme, but we find randomized rounding comes relatively close.

### Availability

Fixseq is freely available for download at http://cgs.csail.mit.edu/fixseq.

## Supporting Information

Figure S1
**Examples of latent** 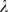 **distributions and mapping function.** Panel (a) shows the latent distribution of log-lambdas for various assays, while panel (b) plots the mapping function for various assays.(EPS)

Figure S2 **Comparison to a covariate-based correction method.** A comparison of rank correlation between replicate experiments is plotted for Fixseq, BEADS, and the two methods run in series. Measurements within each boxplot are computed via bootstrapping.
(EPS)

Table S1 **Comparison to a specialized ChIP-seq event caller.** Correlation in q-value across replicates is shown for a set of hESC CTCF ChIP-seq experiments, with varying count preprocessing schemes.
(PDF)

Table S2 **Analyzed ChIP-seq experiments.** Accession numbers and details for ChIP-seq experiments.
(PDF)

Table S3 **Analyzed RNA-seq experiments.** Accession numbers and details for RNA-seq experiments.
(PDF)

Table S4 **Analyzed DNase-seq experiments.** Accession numbers and details for DNase-seq experiments.
(PDF)

Text S1 **Supplementary methods.** Supplementary results and a description of data sources and processing.
(PDF)

Dataset S1 **Software.** Code, documentation, and test data implementing the Fixseq method.
(ZIP)
