# Supplementary material for: Universal Count Correction for High-Throughput Sequencing
Source: PLoS Comput Biol. 2014 Mar 6;10(3):e1003494. doi: 10.1371/journal.pcbi.1003494 (PMC3945112; doi:10.1371/journal.pcbi.1003494)
Supplement: Table S1 — Comparison to a specialized ChIP-seq event caller. Correlation in q-value across replicates is shown for a set of hESC CTCF ChIP-seq experiments, with varying count preprocessing schemes. (PDF) [file pcbi.1003494.s003.pdf]

Table S1. Comparison to a specialized ChIP-seq event caller

Method	Raw	Deduplication	FIXSEQ	GEM
correlation	0.672	0.675	0.677	0.697

Correlation in q-value across replicates is shown for a set of hESC CTCF ChIP-seq experiments, with varying count preprocessing schemes.
