# Supplementary material for: Universal Count Correction for High-Throughput Sequencing
Source: PLoS Comput Biol. 2014 Mar 6;10(3):e1003494. doi: 10.1371/journal.pcbi.1003494 (PMC3945112; doi:10.1371/journal.pcbi.1003494)
Supplement: Table S2 — Analyzed ChIP-seq experiments. Accession numbers and details for ChIP-seq experiments. (PDF) [file pcbi.1003494.s004.pdf]

Table S2. Analyzed ChIP-seq experiments

Lab	Factor	File(s)	Replicates
Hudson Alpha	Atf2	wgEncodeHaibTfbsH1hescAtf2sc81188V0422111AlnRep*.bam	2
Hudson Alpha	Atf3	wgEncodeHaibTfbsH1hescAtf3V0416102AlnRep*.bam	2
Hudson Alpha	Bcl11a	wgEncodeHaibTfbsH1hescBcl11aPcr1xAlnRep1.bam	1
Hudson Alpha	Bcl11a	wgEncodeHaibTfbsH1hescBcl11aV0416102AlnRep2.bam	1
Hudson Alpha	Ctcf	wgEncodeHaibTfbsH1hescCtcfsc5916V0416102AlnRep*.bam	2
Hudson Alpha	Egr1	wgEncodeHaibTfbsH1hescEgr1V0416102AlnRep*.bam	2
Hudson Alpha	Fosl1	wgEncodeHaibTfbsH1hescFosl1sc183V0416102AlnRep*.bam	2
Hudson Alpha	Gabp	wgEncodeHaibTfbsH1hescGabpPcr1xAlnRep*.bam	2
Hudson Alpha	Hdac2	wgEncodeHaibTfbsH1hescHdac2sc6296V0416102AlnRep*.bam	2
Hudson Alpha	Jund	wgEncodeHaibTfbsH1hescJundV0416102AlnRep*.bam	2
Hudson Alpha	Nanog	wgEncodeHaibTfbsH1hescNanogsc33759V0416102AlnRep*.bam	2
Hudson Alpha	Nrsf	wgEncodeHaibTfbsH1hescNrsfV0416102AlnRep*.bam	2
Hudson Alpha	P300	wgEncodeHaibTfbsH1hescP300V0416102AlnRep*.bam	2
Hudson Alpha	Pol2	wgEncodeHaibTfbsH1hescPol24h8V0416102AlnRep*.bam	2
Hudson Alpha	Pol2	wgEncodeHaibTfbsH1hescPol2V0416102AlnRep*.bam	2
Hudson Alpha	Pou5f1	wgEncodeHaibTfbsH1hescPou5f1sc9081V0416102AlnRep*.bam	2
Hudson Alpha	Rad21	wgEncodeHaibTfbsH1hescRad21V0416102AlnRep*.bam	2
Hudson Alpha	Sin3	wgEncodeHaibTfbsH1hescSin3ak20Pcr1xAlnRep*.bam	2
Hudson Alpha	Six5	wgEncodeHaibTfbsH1hescSix5Pcr1xAlnRep*.bam	2
Hudson Alpha	Sp1	wgEncodeHaibTfbsH1hescSp1Pcr1xAlnRep*.bam	2
Hudson Alpha	Sp2	wgEncodeHaibTfbsH1hescSp2V0422111AlnRep*.bam	2
Hudson Alpha	Sp4	wgEncodeHaibTfbsH1hescSp4v20V0422111AlnRep*.bam	2
Hudson Alpha	Srf	wgEncodeHaibTfbsH1hescSrfPcr1xAlnRep*.bam	2
Hudson Alpha	Taf1	wgEncodeHaibTfbsH1hescTaf1V0416102AlnRep*.bam	2
Hudson Alpha	Taf7	wgEncodeHaibTfbsH1hescTaf7sc101167V0416102AlnRep*.bam	2
Hudson Alpha	Tcf12	wgEncodeHaibTfbsH1hescTcf12Pcr1xAlnRep*.bam	2
Hudson Alpha	Usf1	wgEncodeHaibTfbsH1hescUsf1Pcr1xAlnRep*.bam	2
Hudson Alpha	Yy1	wgEncodeHaibTfbsH1hescYy1sc281V0416102AlnRep*.bam	2
Stanford/Yale	Brca1	wgEncodeSydhTfbsH1hescBrca1IggrabAlnRep*.bam	2
Stanford/Yale	Cjun	wgEncodeSydhTfbsH1hescCjunIggrabAlnRep*.bam	2
Stanford/Yale	Cmyc	wgEncodeSydhTfbsH1hescCmycIggrabAlnRep*.bam	2
Stanford/Yale	Ctbp2	wgEncodeSydhTfbsH1hescCtbp2UcdAlnRep*.bam	2
Stanford/Yale	Jund	wgEncodeSydhTfbsH1hescJundIggrabAlnRep*.bam	2
Stanford/Yale	Max	wgEncodeSydhTfbsH1hescMaxUcdAlnRep*.bam	2
Stanford/Yale	Nrf1	wgEncodeSydhTfbsH1hescNrf1IggrabAlnRep*.bam	2
Stanford/Yale	Rad21	wgEncodeSydhTfbsH1hescRad21IggrabAlnRep*.bam	2
Stanford/Yale	Rfx5	wgEncodeSydhTfbsH1hescRfx5200401194IggrabAlnRep*.bam	2
Stanford/Yale	Suz12	wgEncodeSydhTfbsH1hescSuz12UcdAlnRep*.bam	2
Stanford/Yale	Tbp	wgEncodeSydhTfbsH1hescTbpIggrabAlnRep*.bam	2
Stanford/Yale	Usf2	wgEncodeSydhTfbsH1hescUsf2IggrabAlnRep*.bam	2
Stanford/Yale	Znf143	wgEncodeSydhTfbsH1hescZnf143IggrabAlnRep*.bam	2
Hudson Alpha	Control	wgEncodeHaibTfbsH1hescRxlchPcr1xAlnRep1.bam	1
Hudson Alpha	Control	wgEncodeHaibTfbsH1hescRxlchV0416102AlnRep1.bam	1
Hudson Alpha	Control	wgEncodeHaibTfbsH1hescRxlchV0422111AlnRep1V2.bam	1
Hudson Alpha	Control	wgEncodeHaibTfbsH1hescRxraV0416102AlnRep1.bam	1
Stanford/Yale	Control	wgEncodeSydhTfbsH1hescInputIggrabAln.bam	1
Stanford/Yale	Control	wgEncodeSydhTfbsH1hescInputUcdAln.bam	1
