# Supplementary material for: Universal Count Correction for High-Throughput Sequencing
Source: PLoS Comput Biol. 2014 Mar 6;10(3):e1003494. doi: 10.1371/journal.pcbi.1003494 (PMC3945112; doi:10.1371/journal.pcbi.1003494)
Supplement: Table S3 — Analyzed RNA-seq experiments. Accession numbers and details for RNA-seq experiments. (PDF) [file pcbi.1003494.s005.pdf]

Table S3. Analyzed RNA-seq experiments

Lab	File(s)	Read Details	Replicates
Caltech	wgEncodeCaltechRnaSeqH1hescR1x75dAlignsRep*V2.bam	75bp SE	2
Caltech	wgEncodeCaltechRnaSeqH1hescR1x75dSplicesRep*V2.bam	75bp SE	2
Caltech	wgEncodeCaltechRnaSeqH1hescR2x75Il200AlignsRep*V2.bam	75bp PE	4
Caltech	wgEncodeCaltechRnaSeqH1hescR2x75Il200SplicesRep*V2.bam	75bp PE	4
Caltech	wgEncodeCaltechRnaSeqH1hescR2x75Il400AlignsRep1V2.bam	75bp PE	1
Caltech	wgEncodeCaltechRnaSeqH1hescR2x75Il400SplicesRep1V2.bam	75bp PE	1
CSHL	wgEncodeCshlLongRnaSeqH1hescCellLongnonpolyaAlnRep*.bam	76bp PE	2
CSHL	wgEncodeCshlLongRnaSeqH1hescCellPapAlnRep*.bam	76bp PE	2
CSHL	wgEncodeCshlLongRnaSeqH1hescCytosolLongnonpolyaAlnRep2.bam	76bp PE	1
CSHL	wgEncodeCshlLongRnaSeqH1hescCytosolPapAlnRep2.bam	76bp PE	1
CSHL	wgEncodeCshlLongRnaSeqH1hescNucleusLongnonpolyaAlnRep2.bam	76bp PE	1
CSHL	wgEncodeCshlLongRnaSeqH1hescNucleusPapAlnRep2.bam	76bp PE	1
GIS	wgEncodeGisRnaSeqH1hescCellPapAlnRep1.bam	varying	1
