# Supplementary material for: Universal Count Correction for High-Throughput Sequencing
Source: PLoS Comput Biol. 2014 Mar 6;10(3):e1003494. doi: 10.1371/journal.pcbi.1003494 (PMC3945112; doi:10.1371/journal.pcbi.1003494)
Supplement: Table S4 — Analyzed DNase-seq experiments. Accession numbers and details for DNase-seq experiments. (PDF) [file pcbi.1003494.s006.pdf]

Table S4. Analyzed DNase-seq experiments

Lab	File(s)	Read Details	Replicates
UW	wgEncodeUwDnaseK562AlnRep*.bam	36 SE	2
