# Supplementary material for: Universal Count Correction for High-Throughput Sequencing
Source: PLoS Comput Biol. 2014 Mar 6;10(3):e1003494. doi: 10.1371/journal.pcbi.1003494 (PMC3945112; doi:10.1371/journal.pcbi.1003494)
Supplement: Text S1 — Supplementary methods. Supplementary results and a description of data sources and processing. (PDF) [file pcbi.1003494.s007.pdf]

Text S1

Implicit thresholding of binned per-base counts is a Poisson assumption

Assume we are given per-base counts at base i as c_i and we want to test whether this set of counts was generated from a null distribution λ.

First, we show that a Poisson assumption leads to thresholding based on per-base counts. By the Neyman-Pearson lemma, the only admissible test statistic is the log-likelihood ratio which is:

$$f(c) = \sum_i c_i \left(\log \left(\sum_i c_i \right) - \log(\lambda) \right) - \left(\sum_i c_i - \lambda \right).$$

It is straightforward to see that this function is monotone with respect to $\sum c_i$, which is due to the fact that $\sum c_i$ is the sufficient statistic of the Poisson.

Now we extend this to the converse: thresholding on per-base counts implies a Poisson assumption. Assume we have some admissible procedure which thresholds on the sum of counts $\sum c_i$. By Neyman-Pearson, there exists a log-likelihood f' such that $exp(f'(\sum c_i))$ is the distribution of c under the alternative hypothesis.

This implies the distribution of c is completely determined by the sufficient statistic $\sum c_i$, and it is a standard result that the Poisson is the only distribution with this sufficient statistic [1].

Examples of latent λ distributions and mapping function

To help illustrate the fitted latent distribution over log-lambda, we include the latent probability distributions estimated by Fixseq in Figure S1a. Fixseq accounts for zero-inflation by placing more mass near negative log-lambda values as well as towers through a larger tail on the right. For a perfectly Poisson experiment we would expect to see a single spike at the true log-lambda value.

We also display the set of mapping functions for ChIP-seq, DNase-seq, and RNA-seq as Figure S1b. All three assays display a short and rapid rise that preserves small counts followed by a plateau that remaps large counts to relatively similar values, leading to a soft thresholding effect.

Enhancing covariate based sequence correction

We demonstrate that Fixseq can be used to model residual overdispersion after correction of common sequencing confounders such as GC content and mappability. We ran BEADS using the recommended defaults to create mappability- and GC-corrected windowed read counts (see documentation at http://beads.sourceforge.net). We compared the BEADS output to data corrected only using Fixseq, only BEADS, as well as BEADS output in conjunction with Fixseq, in Figure S2.

RNA-seq exon counting is an ideal test case for covariate count correction, since the assumptions for correcting for mappability and GC content are reasonable and BEADS outputs binned count statistics, which are difficult to use in base-pair resolution methods used in the ChIP-seq and DNase-seq comparisons. The benefits of Fixseq are its universal nature, which does not require additional tuning or modeling for new sequencing assay types, and its creation of processed datasets that are in the same form as the original data, allowing for their use in any downstream processing algorithm.

The results demonstrate that Fixseq provides complementary information to covariate based count correction and enhances the output of BEADS. Therefore even in cases where covariate count correction is more appropriate due to suitability of covariate assumptions and binning, Fixseq still provides benefits via post-correction processing.

Comparison to specialized ChIP-caller (GEM)

We compared Fixseq to an assay-specific adaptive overdispersion correction heuristic developed for a sequence-aware ChIP-seq caller [2].

A total of four cases were tested on human h-ESC CTCF ChIP-seq as an example of an well-established quality ChIP experiment and we measured the replicate correlation in q-value. The results are shown in Table S1.

Datasets

All ChIP-seq and RNA-seq experiments for the h1-ESC cell line and DNAse-seq experiments for the K562 cell line outside the publication embargo window were identified from the ENCODE website at http://www.encodeproject.org/ENCODE/dataMatrix/encodeDataMatrixHuman.html. Aligned read files for each experiment were downloaded as BAM files from http://hgdownload.cse.ucsc.edu/goldenPath/hg19/encodeDCC. Table S2 lists the ChIP-seq experiments analyzed in this work, Table S3 lists the RNA-seq experiments, and Table S4 lists the DNAse-seq experiments. Only experiments with available replicates were analyzed in consistency comparisons.

Read analysis pipeline

The DNAse analysis software CENTIPEDE was downloaded from http://centipede.uchicago.edu.

ChIP-seq datasets were analyzed by first matching each signal run to the corresponding lab-specific input control experiment. When multiple control experiments were available, they were pooled at the read level (following the ENCODE workflow described in the "Reproducibility and automatic thresholding of ChIP-seq data" section at http://encodeproject.org/ENCODE/encodeTools.html).

Peaks were called using two of the three preferred ENCODE event callers, MACS and PeakSeq. MACS version 2.0.10.20120913 was downloaded from https://github.com/taoliu/MACS/ and PeakSeq version 1.1 was downloaded from http://info.gersteinlab.org/PeakSeq. MACS was called with the flags `-g hs -q 0.01 --keep-dup all --to-large`. PeakSeq was run with a `target_FDR` of 0.01 and `max_Qvalue` of 0.01. For all other settings, the default parameters were used.

For the RNA-seq data, exon counts were obtained by analyzing the BAM read mapping files with the `bedtools multicov` command (BEDTools version 2.16.2). CCDS exon annotations were obtained from the UCSC Genome Browser (http://hgdownload.cse.ucsc.edu/goldenPath/hg19/database/ccdsGene.txt.gz). For exon sums using reweighted read counts, we modified the bedtools package to use per-read weights in its exon-level sums.

For read rescaling comparisons, including de-duplication and Fixseq rescaling, modified datasets were constructed when streaming read input data to the respective processing algorithms.

References

[1] Casella, G., Berger, R.L.: Statistical inference, vol. 70. Duxbury Press Belmont, CA (1990)

[2] Guo, Y., Mahony, S., Gifford, D.K.: High resolution genome wide binding event finding and motif discovery reveals transcription factor spatial binding constraints. PLoS Computational Biology 8(8), e1002638 (2012)
