# Supplementary material for: Universal Count Correction for High-Throughput Sequencing
Source: PLoS Comput Biol. 2014 Mar 6;10(3):e1003494. doi: 10.1371/journal.pcbi.1003494 (PMC3945112; doi:10.1371/journal.pcbi.1003494)
Supplement: Dataset S1 — Software. Code, documentation, and test data implementing the Fixseq method. (ZIP) [file pcbi.1003494.s008.zip › thashim-fixseq-b7f0e45600be/llhfit.pdf]

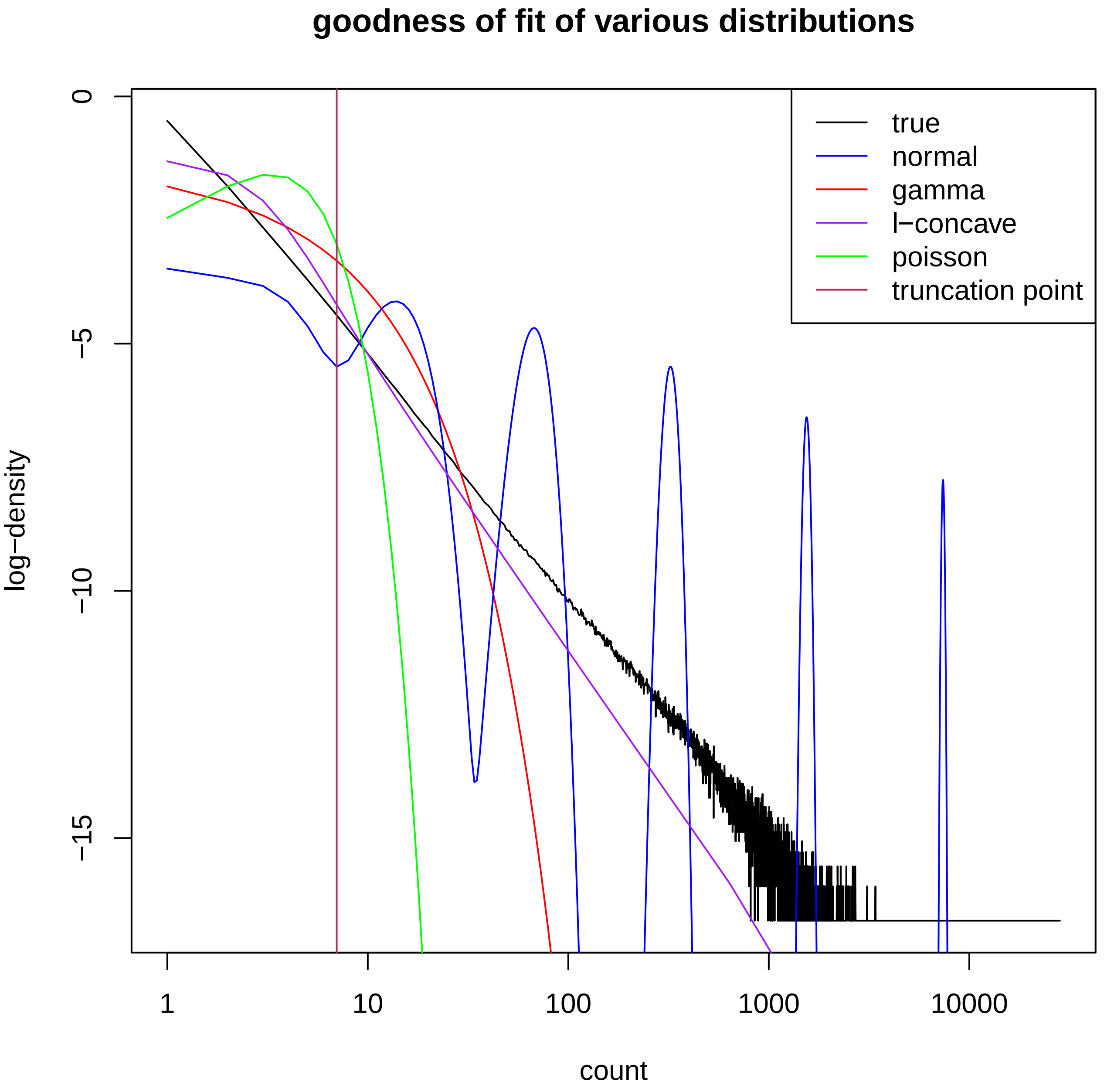
goodness of fit of various distributions
log−density
count
0
−5
−10
−15
1
10
100
1000
10000
true
normal
gamma
l−concave
poisson
truncation point

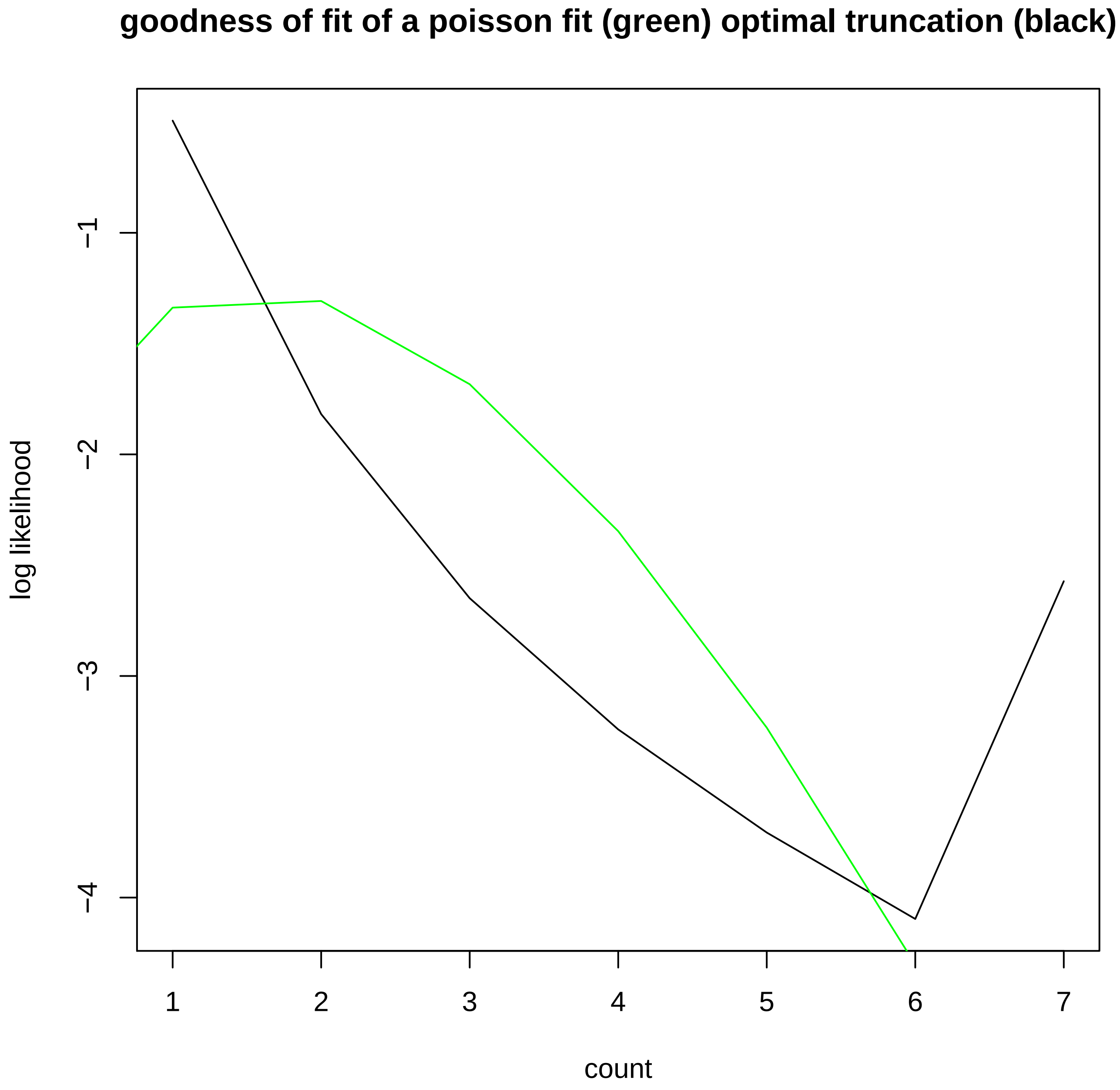
goodness of fit of a poisson fit (green) optimal truncation (black)
log likelihood
−1
−2
−3
−4
1
2
3
4
5
6
7
count

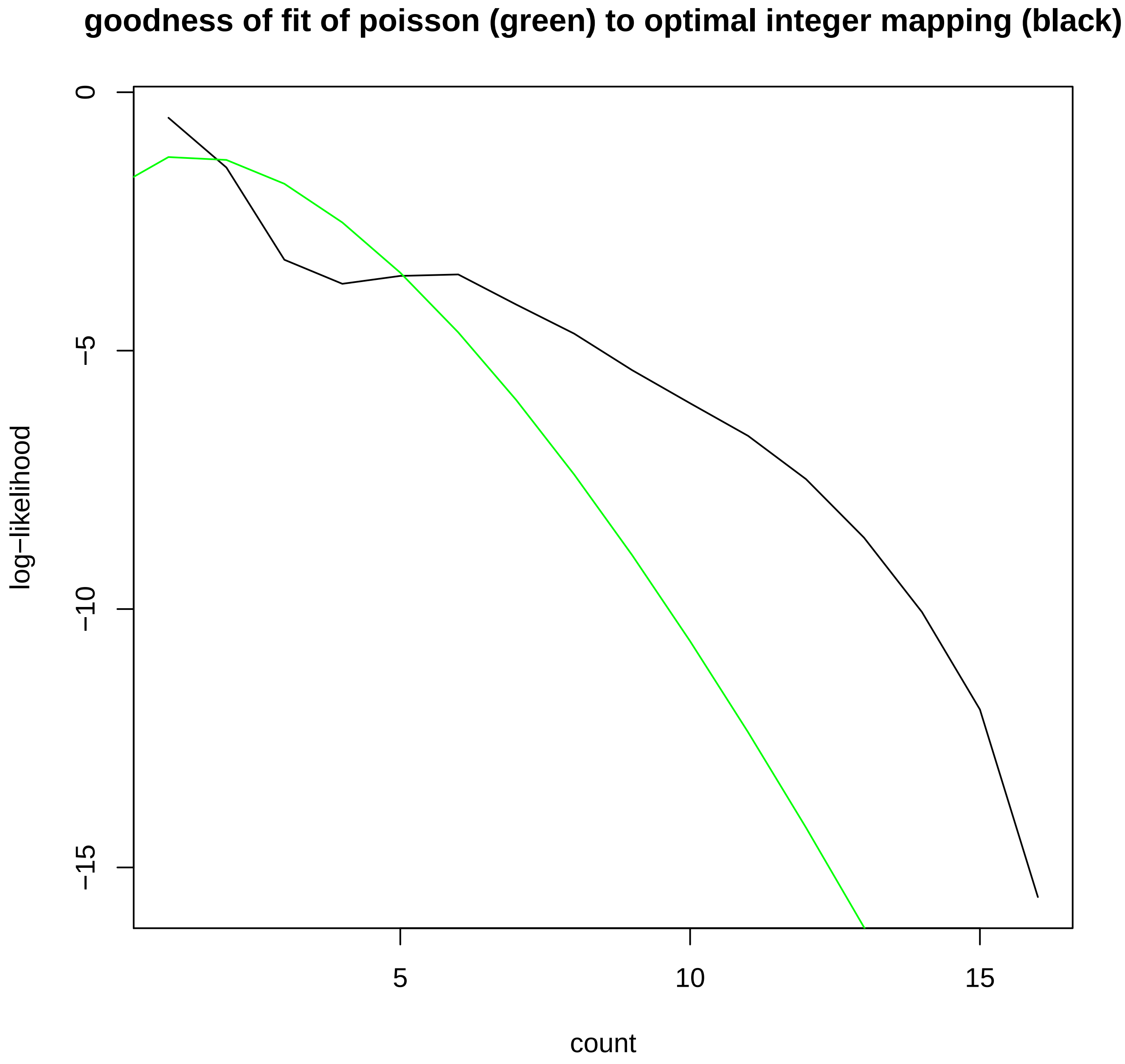
goodness of fit of poisson (green) to optimal integer mapping (black)
0
−5
−10
−15
log−likelihood
5
10
15
count
