# Supplementary figures and images for: Universal Count Correction for High-Throughput Sequencing
Source: PLoS Comput Biol. 2014 Mar 6;10(3):e1003494. doi: 10.1371/journal.pcbi.1003494 (PMC3945112; doi:10.1371/journal.pcbi.1003494)

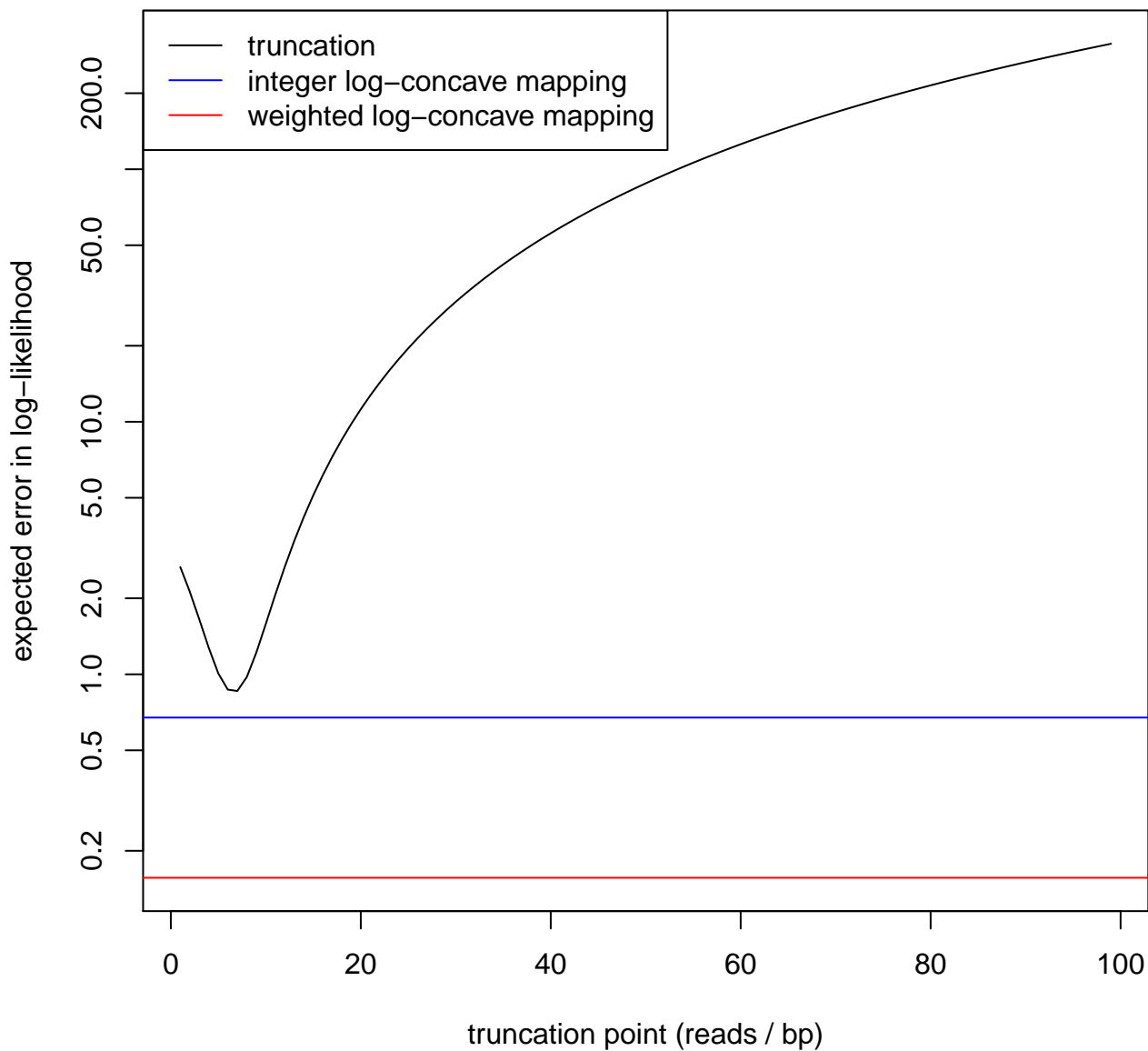

Supplement: Dataset S1 — Software. Code, documentation, and test data implementing the Fixseq method. (ZIP) [file pcbi.1003494.s008.zip › thashim-fixseq-b7f0e45600be/truncation-loss.pdf]
